# MicroRNA-760 inhibits cell proliferation and invasion of colorectal cancer by targeting the SP1-mediated PTEN/AKT signalling pathway

**DOI:** 10.3892/mmr.2022.12783

**Published:** 2022-06-27

**Authors:** Xiaoyan Li, Yuansheng Ding, Naiqing Liu, Qinli Sun, Jie Zhang

Mol Med Rep 16: 9692–9700, 2017; DOI: 10.3892/mmr.2017.7814

Following the publication of this paper, it was drawn to the Editors’ attention by a concerned reader that certain of the cell migration and invasion assay data shown in Figs. 2C and 5C were strikingly similar to data appearing in different form in other articles by different authors. Owing to the fact that the contentious data in the above article had already been published elsewhere, or were already under consideration for publication, prior to its submission to *Molecular Medicine Reports*, the Editor has decided that this paper should be retracted from the Journal. The authors were asked for an explanation to account for these concerns, but the Editorial Office did not receive a satisfactory reply. The Editor apologizes to the readership for any inconvenience caused.

